# Extensive genome introgression between domestic ferret and European polecat during population recovery in Great Britain

**DOI:** 10.1093/jhered/esac038

**Published:** 2022-08-06

**Authors:** Graham J Etherington, Adam Ciezarek, Rebecca Shaw, Johan Michaux, Elizabeth Croose, Wilfried Haerty, Federica Di Palma

**Affiliations:** Organisms and Ecosystems, The Earlham Institute, Norwich Research Park, Norwich, United Kingdom; Organisms and Ecosystems, The Earlham Institute, Norwich Research Park, Norwich, United Kingdom; Organisms and Ecosystems, The Earlham Institute, Norwich Research Park, Norwich, United Kingdom; Department of Life Sciences, University of Liège, 4000 Liège, Belgium; The Vincent Wildlife Trust, Ledbury, Herefordshire, United Kingdom; Organisms and Ecosystems, The Earlham Institute, Norwich Research Park, Norwich, United Kingdom; Organisms and Ecosystems, The Earlham Institute, Norwich Research Park, Norwich, United Kingdom

**Keywords:** conservation, domestic ferret, European polecat, genomics, introgression, Mustelids

## Abstract

The European polecat (*Mustela putorius*) is a mammalian predator which occurs across much of Europe east to the Ural Mountains. In Great Britain, following years of persecution the range of the European polecat contracted and by the early 1900s was restricted to unmanaged forests of central Wales. The European polecat has recently undergone a population increase due to legal protection and its range now overlaps that of feral domestic ferrets (*Mustela putorius furo*). During this range expansion, European polecats hybridized with feral domestic ferrets producing viable offspring. Here, we carry out population-level whole-genome sequencing on 8 domestic ferrets, 19 British European polecats, and 15 European polecats from the European mainland. We used a range of population genomics methods to examine the data, including phylogenetics, phylogenetic graphs, model-based clustering, phylogenetic invariants, ABBA-BABA tests, topology weighting, and Fst. We found high degrees of genome introgression in British polecats outside their previous stronghold, even in those individuals phenotyped as “pure” polecats. These polecats ranged from presumed F1 hybrids (gamma = 0.53) to individuals that were much less introgressed (gamma = 0.2). We quantify this introgression and find introgressed genes containing Fst outliers associated with cognitive function and sight.

## Introduction

Mustelidae form the largest family of the order Carnivora, comprising around 60 species. *Mustela*, a genus of Mustelidae that originated at least 5.3 MYA, contains around 17 species of weasels, stoats, mink, polecats, and ferrets (Koepfli *et al.* 2008). The European polecat (*Mustela putorius*) occurs in a range of habitats including farmland, wetland, and woodland, where it feeds mainly on rabbits, rodents, amphibians, and small birds (Lodé 1997; Croose 2016). The domestic ferret (*M. putorius furo*) has long been associated with man and is thought to have been domesticated around 2000 years ago and introduced in Great Britain around the 11th century (Thomson 1951). The ancestral species to the ferret is also disputed with many authors treating the domestic ferret as a descendant of the European polecat, but others treating it as a descendant of the Steppe polecat (*M. eversmanii*) (e.g. Blandford and Walton 1991; Driscoll *et al.* 2009), or their findings being inconclusive between the 2 potential ancestral species (Davison *et al.* 1999; Kurose *et al.* 2008). European polecats are known to hybridize with a number of other mustelid species, including Steppe polecat (Cserkész *et al.* 2021; Szatmari *et al.* 2021), European Mink (Lode *et al.* 2005; Cabria *et al.* 2011), and domestic ferret (Davison *et al.* 1999; Costa *et al.* 2012).

**Fig. 1.**
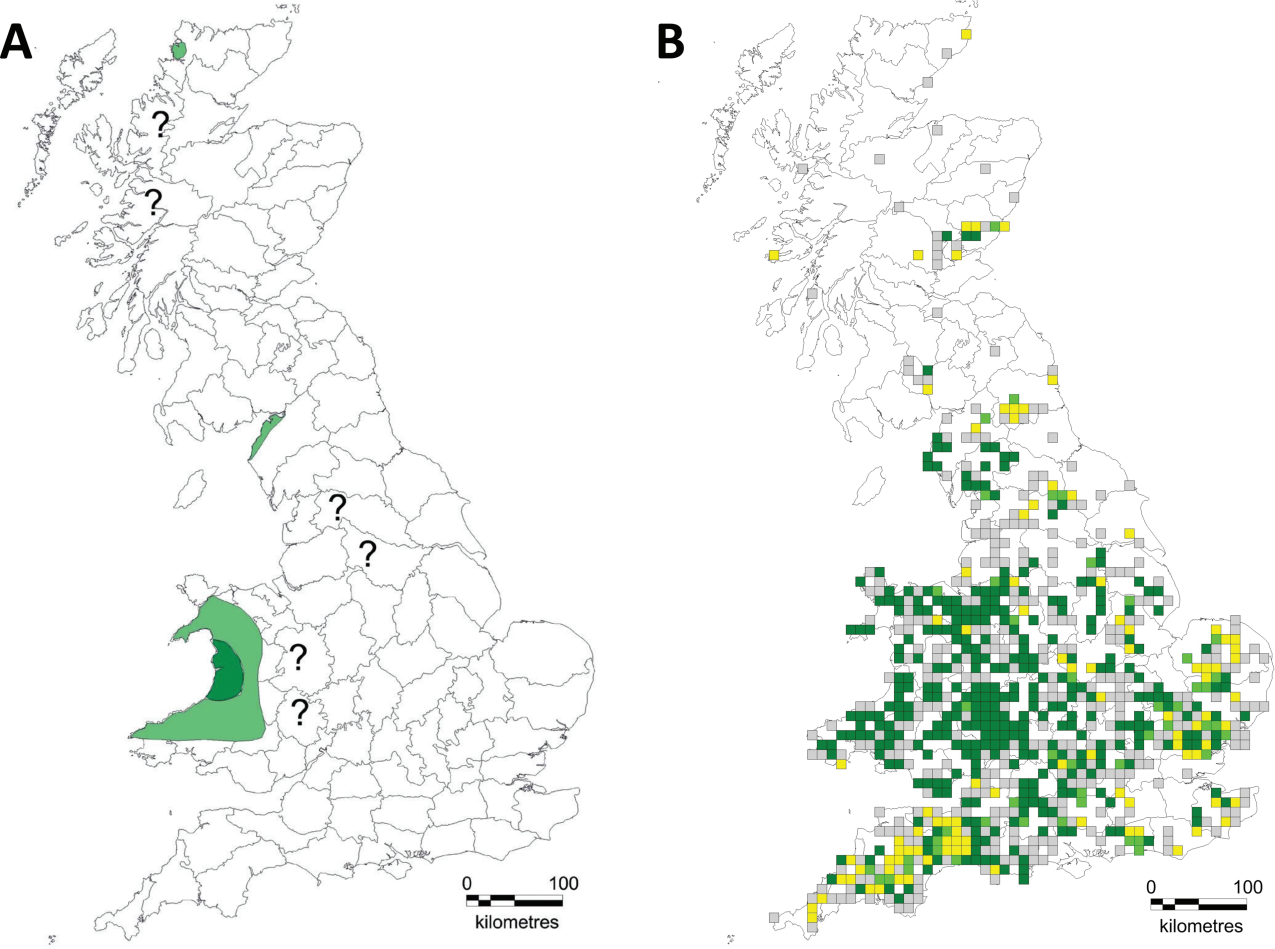
(A) The range of the European polecat across the Great Britain in 1915. Dark green indicates stronghold, pale green indicates localized or rare occurrences, and “?” indicates uncertain status (Langley and Yalden 1977). (B) The distribution of hectads (10 km × 10 km squares) in which verifiable records of true polecats (dark green), polecat–ferrets (yellow), both true polecats and polecat–ferrets (lime green) and unverifiable records (gray) were received during the 2014 to 2015 European polecat survey (Croose 2016).

In Great Britain, the European polecat (hereafter “polecat”) has a chequered history. At the start of the 1800s, it was widespread across almost the whole of Great Britain, but from the mid-1800s it was systematically persecuted to near extinction. By the end of the century it became confined mainly to small areas of central Wales and English counties bordering Wales (Fig. 1A) (Langley and Yalden 1977; Croose 2016). Remnant populations remained in some areas, particularly in parts of northern Scotland and Cumbria, but these populations likely disappeared by the mid 1900s (Birks 2015). Following legal protection and a reduction in persecution, polecat numbers started to increase from the 1930s, seeing a range expansion that now reaches almost every county in England and Wales along with limited areas in Scotland (Fig. 1B) (Croose 2016).

Genome introgression through hybridization involving endangered species is a conservation issue because it can lead to the genetic extinction of the endangered species. Whether it be from closely related wild taxa or domesticated species, through introduction or range expansion, the negative consequences of introgression can include outbreeding depression and swamping of the genome in the endangered species (Marshall and Spalton 2000; Marr *et al.* 2002) and can lead to the genetic extinction of the endangered species (Rhymer and Simberloff 1996). A well-known example of this is the Scottish wildcat, which is now highly introgressed with domestic cat to the point where most wild-living wildcats have evidence of hybridization, with the “purest” wildcats being found in captive breeding programs (Hubbard *et al.* 1992; Montague *et al.* 2014; Nussberger et al. 2014, 2018; Oliveira *et al.* 2015; Breitenmoser *et al.* 2019).

During their range expansion, polecats hybridized with feral domestic ferrets most frequently at the edge of the polecats’ range (Costa *et al.* 2013; Croose 2016). Previous studies focusing on polecat mitochondrial haplotypes reported between 2 (1 ferret and 1 polecat [Davison *et al.* 1999]) and 3 (1 ferret and 2 polecat) (Costa *et al.* 2013) haplotypes occurring in Great Britain. Further work using 11 microsatellite loci suggested genome introgression was quite prevalent, occurring in 31% of polecat samples. However, due to an insufficient number of microsatellite loci, no F1 hybrids or backcrossed individuals could be identified (Costa *et al.* 2013). Polecats are difficult to phenotypically identify, with many individuals displaying a polecat phenotype but possessing ferret haplotypes (Birks and Kitchener 1999; Costa *et al.* 2013).

Previous work on British polecats has concentrated mainly on the analyses of mitochondrial genes or used a small number of microsatellites that have lacked the power to quantify the extent of per-individual genome introgression. There are no studies that look at adaptive introgression in polecats and the amount of research relating polecat and ferret genotypes to phenotype is minimal. With the advent of next-generation sequencing and the development of new population genomics tools, it is now possible to identify introgression between populations and within individuals at almost base-pair resolution. Here, we carry out population-level whole-genome sequencing on a range of domestic ferrets, polecats from the European mainland, and polecats and polecat–ferret hybrids from Great Britain and examine the extent of domestic ferret introgression in British polecats.

## Methods

### Sample origins

In total we analyzed 49 different samples of mustelid. We sourced 37 roadkill *Mustela* samples, which comprised 15 European polecats from the European mainland (2 from Spain, 3 from Austria, 3 from France, 5 from Italy, and 2 from Germany), 16 European polecats phenotyped as “pure” and 3 phenotyped as polecat–ferret hybrids from Great Britain, 2 Steppe polecats (*M. eversmanii*), and 1 Least weasel (*M. nivalis*), along with 8 domestic ferrets from The Broad Institute (Supplementary Table S1). The British polecat samples were further subdivided by location, with 6 samples from Wales (or counties bordering Wales and England) and the remaining 13 samples allocated to the “English” group (10 pure and 3 hybrids). Additionally, 4 Black-footed ferrets (*M. nigripes*) whole-genome sequences were obtained from publicly available data (PRJNA25445).

### Sample preparation and sequencing

DNA extraction was carried out using Qiagen DNeasy Blood and Tissue following the manufacture’s protocol. Samples were quality checked using the Nanodrop Spectrophotometer. Illumina short-read sequencing was carried out on all samples (Supplementary Table S2). The full protocol is described under “Library construction protocols” at protocols.io under dx.doi.org/10.17504/protocols.io.bww9pfh6.

The 8 domestic ferrets represent the most diverse set from 76 other ferret samples and were selected as follows. A Nimblegen capture array was designed, targeted to 2 Mb from each of the 6 largest scaffolds from the ferret genome assembly for a total of 12 Mb (Peng *et al.* 2014). All 76 samples used in the capture were drawn from multiple populations (United States, China, and Australia) and phenotypes (longhair, sable, albino, and cinnamon). The capture library was then sequenced on an Illumina HiSeq and GATK was then used to call single-nucleotide polymorphisms (SNPs) (McKenna *et al.* 2010). Clustering and phylogenetic methods were then used to select 8 samples that represented the most diverse set from the original 76. These 8 samples were used for whole-genome sequencing. The capture array was also used further in this work to calculate a false discovery rate (FDR) in our “Allele Sharing” method below.

### Mitochondrial genome assembly

For all 49 samples, we used the program Mitoz “all” (Meng *et al.* 2019) to filter the genomic reads for those of a mitochondrial origin, assemble them de novo, identify mitochondrial scaffolds, and annotate each scaffold. Using the European polecat mitochondrial genome (accession NC_020638.1) as an anchor, assemblies were then reordered so they all started at the same relative positions. We then removed sites containing gaps using Jalview (v2.11.2.2) (Waterhouse *et al.* 2009). After alignment and gap removal, the mitochondrial genome alignment for all 49 samples measured 15,507 base pairs. The full protocol is described under “Mitochondrial genome assembly” at protocols.io under dx.doi.org/10.17504/protocols.io.bqzbmx2n.

### Genomic variant calling and consensus assembly

Read quality was assessed using the FASTQC tool (Andrews 2010). Reads with low-quality scores (<*Q*20) were trimmed using the Trim Galore! tool (Krueger 2015). Next, we assembled consensus genome sequences for each sample, following the Genome Analysis Toolkit (GATK, version 4.1.3) best-practice workflow for SNP and Indel calling in nonmodel organisms (Poplin *et al.* 2017), which is fully described under “GATK Nuclear variant discovery and consensus assembly” at protocols.io under dx.doi.org/10.17504/protocols.io.bqzgmx3w.

Briefly, reads were mapped using BWA MEM (Li and Durbin 2009) to a new version of the domestic ferret reference genome MusPutFur1.0 scaffolded with Bionano data (accession GCA_920103865). After marking duplicates with Picard Tools (Broad_Institute 2019), variants were called using the GATK HaplotypeCaller (using the PCR-free indel model where appropriate). The mean value of SNP quality, read depth, and mapping quality (QUAL, DP, and MQ values respectively from the VCF file) was calculated and GATK VariantFiltration was carried out marking SNPs with values lower than the mean scores as failing the filter. Using the variants that passed filtering, a recalibration table was created using GATK BaseRecalibrator and then Base Quality Score Recalibration was carried out using the table with the GATK ApplyBQSR tool to produce a recalibrated BAM file. This was then followed by a second round of GATK HaplotypeCaller using the recalibrated BAM file outputting a GVCF file (all sites, regardless of haplotype). Next, GATK GenomicsDBImport was used to import all GVCF files from all samples, followed by GATK GenotypeGVCFs to create a single multisample VCF file, followed by the creation of a SNP-only version using the GATK SelectVariants tool. Finally, consensus genome sequences were created for each sample using the GATK FastaAlternateReferenceMaker tool.

### Genome-wide SNP data

Whole-genome SNPs for all 49 samples were filtered using PLINK v1.9 with an LD threshold of 0.8, within 5 kb windows. (Chang *et al.* 2015). This subset of SNPs was further filtered using bcftools (Danecek and McCarthy 2017) to only include sites with at least 3 alternate alleles and which contained at least 1 homozygous alternate allele and 1 homozygous reference allele in order to allow RAxML-NG to be run with a model with ascertainment-bias correction (Kozlov *et al.* 2019). This resulted in 2,694,963 SNPs. These sites were then reformatted into phylip format for input into RAxML-NG.

### Phylogenetic analyses

#### RAxML

We used ClustalW to align the 49 de novo mitochondrial genome assemblies (Thompson *et al.* 2002). We then used RAxML-NG to construct maximum likelihood (ML) phylogenies for the de novo mitochondrial genome sequences and the genome-wide SNP data (Kozlov *et al.* 2019). We ran RAxML in 2 stages. The first is the “check” and “parse” options of RAxML. “check” ensures for alignment correctness and removes empty columns. “parse” creates a binary MSA file (named *raxml.rba). It also estimates the resources (cores and memory) needed to run RAxML efficiently. Using the resources advised by the previous step, we ran raxml “all” which runs a ML tree search and nonparametric bootstrap by resampling alignment columns and reinferring a tree for each bootstrap replicate. The ML tree search was started with 20 distinct trees and bootstrapping analysis was started with 1,000 replicates. Full details are described under Step in “Population Structure Phylogenetics” at protocols.io under dx.doi.org/10.17504/protocols.io.bretm3en.

#### TreeMix

We also used TreeMix to examine historical relationships among 7 predefined populations containing all 49 samples—domestic ferret, European mainland polecats, Welsh polecats, English polecats, Steppe polecats, Black-footed ferrets, and Least weasel (outgroup) (see “Analyses populations” in Supplementary Table S1 for sample-specific population allocations) (Pickrell and Pritchard 2012). TreeMix uses allele frequency datasets to estimate relationships among populations, using a graph representation that allows both population splits and migration events (or “edges”—branches of a tree that poorly fit a tree-like structure, inferring gene flow between populations). The contribution of each donor population is weighted. These weights relate to the proportion of alleles in the descendant population that originated in each donor population.

Using the LD-pruned SNPs described above, we removed missing data using VCFTOOLS and ran TreeMix under models allowing between 1 and 5 migration edges, with 500 bootstrapping replicates, using Least weasel as the outgroup. To estimate the optimal number of migration edges, we used the R package OptM using the default Evanno method (Fitak 2021). The number of migration edges was chosen based on a plateau of log likelihoods and when greater than 99.8% of the variance was explained. Full details are described under Step 2 in “Population Structure and Phylogenetics” at protocols.io under dx.doi.org/10.17504/protocols.io.bretm3en.

#### Population structure

We used model-based clustering implemented in ADMIXTURE to visualize the genetic ancestry of the 42 samples of domestic ferrets and European polecats. ADMIXTURE calculates ML estimation of individual ancestries from multilocus SNP genotype datasets and models the data over a given number of ancestral populations (“*K*”). We separated the samples into 5 populations, domestic ferrets, mainland European polecats, Welsh polecats, English polecats, and hybrid polecats. After selecting samples with GATK SelectVariants, we used Plink to filter missing genotypes (at a frequency of 0.999) and to reformat our data into the BED format required by ADMIXTURE. We ran ADMIXTURE using “*K*” values between 2 and 5 and included the cross-validation option (--cv) to estimate the most likely value of “*K*.” A good value of *K* will exhibit a low cross-validation error compared with other *K* values. A maximum of *K* = 5 was used as this allowed the identity of ancestral populations outside of the 3 main defined populations of domestic ferret, Welsh polecats, and mainland European polecats in the analysis.

Our output was visualized using Pong (https://github.com/ramachandran-lab/pong). Full details are described under Step 3 in “Population Structure and Phylogenetics” at protocols.io under dx.doi.org/10.17504/protocols.io.bretm3en.

#### Nucleotide diversity

Given that Great Britain (and hence any existing populations of polecats) has been isolated from the European mainland for around 8,000 years and that the polecat it has undergone a recent population bottleneck, we examined whole-genome nucleotide diversity (*π*) for Welsh polecats and compared it to polecats from mainland Europe, domestic ferret, and 2 of our outgroups (Black-footed ferret and Steppe polecat). Nucleotide diversity was calculated using VCFTOOLS over 100 kb windows and then calculating the mean score over all of the windows.

### Introgression

#### HyDe analysis

HyDe is a software package that detects hybridization in multiple sequence alignments using site pattern probabilities and phylogenetic invariants. Hyde utilizes phylogenetic invariants and uses a model that considers both coalescence and hybridization together (Cavender and Felsenstein 1987; Blischak *et al.* 2018; Kubatko and Chifman 2019).

The consensus genome sequences for each sample were concatenated into a single fasta sequence, which were then in turn concatenated to construct a single multiple sequence alignment containing all samples. Variant sites were then extracted using SNP sites (Page *et al.* 2016). We partitioned 43 samples into 5 populations—domestic ferrets, mainland European polecats, Welsh polecats, English polecats, along with weasel as the outgroup. Phenotypic hybrids were not treated as a separate case in order to make no prior assumptions on genetic backgrounds assigned only by phenotype (which is notoriously difficult to assign correctly). HyDe was used to run the “full” analysis (using HyDe’s “run_hyde.py” script), which calculates site pattern probabilities for each population and tests all combinations of 3 populations in all directions for evidence of hybridization (with the addition of the weasel outgroup in each test). Hyde filters the results from the hybridization detection analysis to only include trios with significant results and where Gamma is between 0 and 1. “Gamma” is an indication as to the proportion of genetic loci contributing from P1 and P2, where a value of 0.5 would indicate a 50:50 hybrid. At the population level, if we are testing 4 individuals in a putative hybrid population and only 2 of them are 50:50 hybrids (gamma = 0.5 for each), then the value of gamma for the whole population will be 0.25.

All individual samples within the population trios included in the significant results were then analyzed in turn (using HyDe’s “individual_hyde.py” script) to obtain individual per-sample values of Gamma. In this scenario, site pattern probabilities are calculated from each “parental” population (i.e. P1, P2, and outgroup), whilst each individual in the putative hybrid population is tested 1 at a time. This allows us to see the variation of individuals in our hybrid population.

Full details are described under Step 1 of “Introgression” at protocols.io under dx.doi.org/10.17504/protocols.io.bq7tmznn.

#### Dsuite analysis

Dsuite is a software tool which is complementary to HyDe in that it searches for introgression and gene flow between populations. It differs from HyDe in that it can be used to examine sliding genomic windows and that it uses ABBA-BABA tests and not phylogenetic invariants. ABBA-BABA tests (also known as Patterson’s *D*-statistic) are commonly used to assess evidence of gene flow by examining patterns of allele sharing between populations or species in genomic datasets (Durand *et al.* 2011). Dsuite is a software package that implements ABBA-BABA tests between designated populations, as well as a number of other closely related methods (Malinsky *et al.* 2020). First, we filtered our data to only included biallelic SNPs using GATK SelectVariants (using the parameters “--restrict-alleles-to BIALLELIC -select-type SNP”). We then used the Dsuite “Dtrios” program to calculates the *D*-statistic for all possible population trios, using the same population partitions used in the HyDe analysis above. We also included the option to use a phylogenetic tree to specify the relationships between populations (see Supplementary Data tree.nwk). Full details are described under Step 2 of “Introgression” at protocols.io under dx.doi.org/10.17504/protocols.io.bq7tmznn.

#### Allele sharing

In order to quantify the extent of introgression in each genome we identified sites at which all samples of domestic ferrets were homozygous to a common allele and in which European polecats from the European mainland where homozygous to a common alternative allele at the same site (using GATK SelectVariants). For this we used all 8 domestic ferret genomes (all of which had a minimum read coverage of 18.9×) and compared them to 8 high-coverage (>10×) European mainland polecats. One sample (euro_LIB21977) was later removed from the analyses due to the suggestion of a small amount of genome introgression present in this sample (Fig. 5). We restricted our analysis to biallelic SNP sites and then summarized our results (using GATK VariantsToTable) to confirm that all the homozygous European polecat sites were alternate to the same allele. Next, we calculated the mean SNP quality score (QUAL) for each site and filtered out any sites with a quality score less than 30. We then took each British polecat sample in turn, identified the genotype at each corresponding site and calculated the proportion of the genomes that had ferret- or polecat-specific alleles. Using the Office of National Statistics (ONS) shapefile for NUTS2 (https://geoportal.statistics.gov.uk/datasets/48b6b85bb7ea43699ee85f4ecd12fd36_0) we allocated each sample to one of the 40 different NUTS2 geographical regions and calculated the mean contribution of ferret- and polecat-specific alleles to polecat genomes in that region.

To calculate the FDR of the allele sharing method, we took the 12 Mb capture array for the 76 domestic ferret samples (see Methods: Samples and Sequencing) and calculated genotypes at each of the fixed positions. We then identified the accuracy of our method by calculating the number of genotype calls in the 76 samples that agreed/disagreed with our ferret-specific allele set. Using GATK SelectVariants, we noted the polecat–ferret fixed sites over the coordinates of the 12 Mb capture array, mapped reads from the 76 capture array samples to the 12 Mb capture array reference and used GATK to call SNPs, restricting the calls to the polecat–ferret fixed sites, following the same method used for the whole-genome analyses above. Full details of the allele sharing protocol and FDR analyses are described under Step 3 of “Introgression” at protocols.io under dx.doi.org/10.17504/protocols.io.bq7tmznn.

#### Topology weighting analysis

Due to variation in lineage sorting and introgression, phylogenetic relationships between populations vary across the genome. In order to highlight regions of the genome where the English polecat is more closely related to the domestic ferret than other wild polecats, we carried out a topology weighting analysis using TWISST (Martin and Van Belleghem 2017). Biallelic SNPs present in all British and mainland European polecats, domestic ferrets, and weasel (43 samples) with missing base calls for less than 5% missing taxa were extracted using bcftools (Danecek and McCarthy 2017), and then phased and imputed using beagle v4.1 with a window size of 10 kb and overlap of 1 kb (Browning and Browning 2007). Phylogenetic trees were then inferred across the longest 23 scaffolds (all at least 25 Mb in length) as well as any where there is putative evidence of adaptive introgression (see methods below). IQTREE v1.6.12 (Nguyen *et al.* 2015) was used to infer trees for each 50 bp window (with at least 40 sites per individual), with an overlap of 10 bp, using scripts adapted from genomics_general (https://github.com/simon_hmartin/genomics_general). Automatic model selection (Kalyaanamoorthy *et al.* 2017) was carried out for each window with ascertainment-bias correction. Topology weighting analysis was then carried out using TWISST (Martin and Van Belleghem 2017). The weightings were plotted in R (v3.5.2), after smoothing with a loess span of 0.05.

#### Adaptive introgression

Fst outliers are often used on SNP data to detect selection (Wright 1951; Weir and Cockerham 1984; Excoffier *et al.* 2009), whilst the *D*-statistics (and its variants) is a frequently used method for detecting ancient admixture events (Green *et al.* 2010). We used the intersection of Fst outliers and genomic loci with significantly high *D*-statistic to identify introgressed regions from domestic ferret under selection in English polecats. We took the most likely trio of populations showing introgression (identified from our previous analyses) and filtered the input for scaffolds longer than 1 Mb where all samples had called genotypes. We then used Dsuite Dinvestigate to identify sliding windows of 1,000 SNPS and their associated admixture statistics (*D*, *f*_d_, *f*_dM_, and df) and selected the windows with the top 1-percent of *f*_dM_ values (Malinsky *et al.* 2020). The *f*_dM_ statistic (a modified version of the *f*_d_ statistic) is symmetrically distributed around zero under the null hypothesis of no introgression and can equally quantify shared variation between P3 and P2 (positive values) or between P3 and P1 (negative values).

Using VCFTools, we then identified the location of the top 1-percentile Fst outliers between the 2 “parental” populations of the introgressed trio (Danecek *et al.* 2011). We filtered down the Fst outliers to those that overlapped with the 1-percentile *f*_dM_ windows and then identified which of these intersections overlapped with genes in the domestic ferret genome annotation (Peng *et al.* 2014).

## Results

### Phylogenetic analyses

**Fig. 2.**
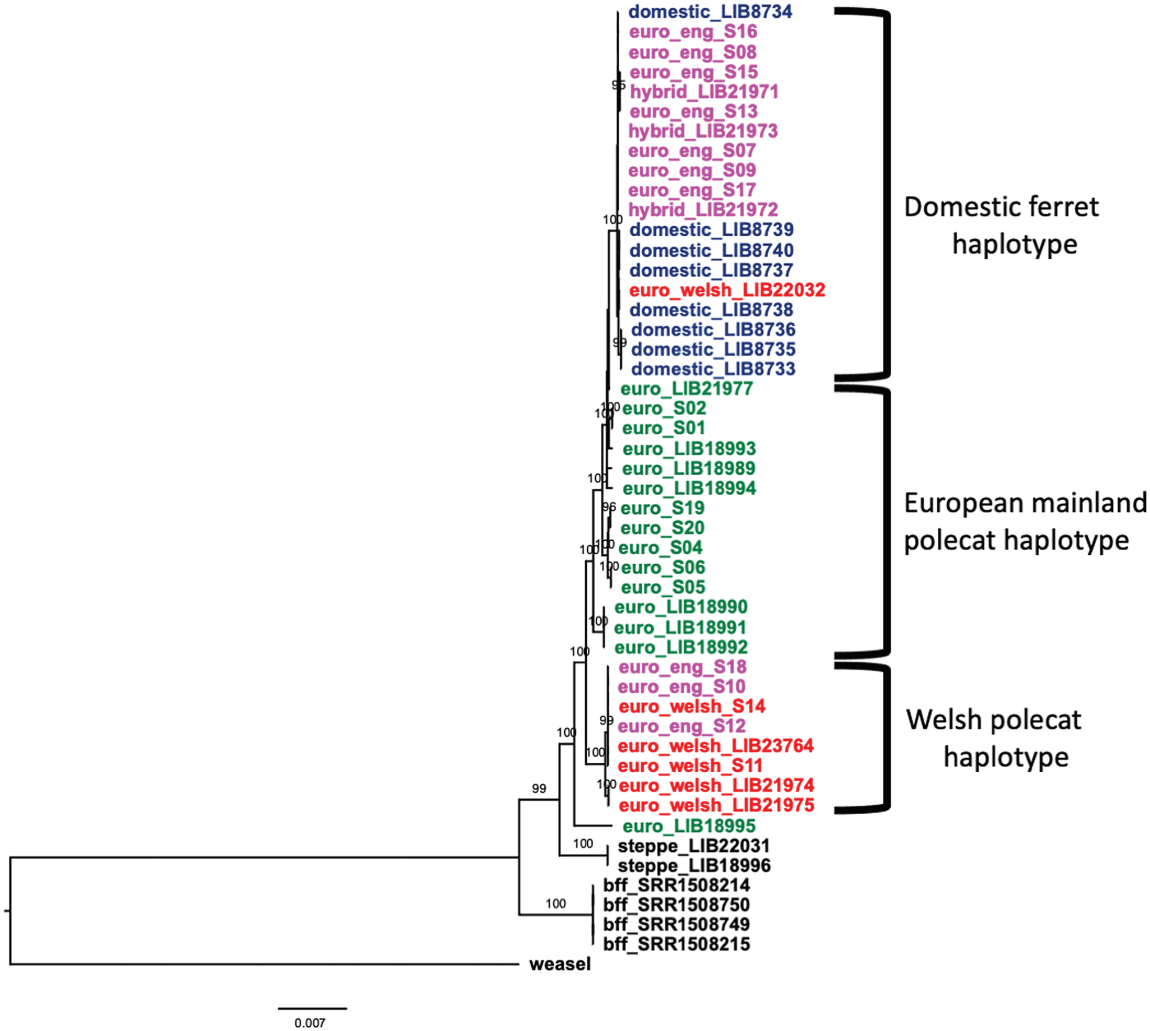
ML phylogeny with 1,000 bootstrap replicates, for whole mitochondrial genomes of all 49 samples in this study, with main haplotype groups annotated. Numbers at nodes refer to bootstrap support values of 95 and above. Domestic ferret samples are labeled as “domestic_” and colored dark-blue, European polecats from mainland Europe are labeled as “euro_” and colored green, European polecats from England are labeled as “euro_eng_” and colored purple, European polecats from Wales are labeled as “euro_welsh_” and colored red, and samples identified (by phenotype) as ferret × polecat hybrids are labeled as “hybrid_” and colored purple (to reflect their English origin). Steppe polecats and Black-footed ferrets are labeled as “steppe_” and “bff_,” respectively, and colored black. The tree is rooted with Least Weasel and branch lengths are in expected substitutions per site.

**Fig. 3.**
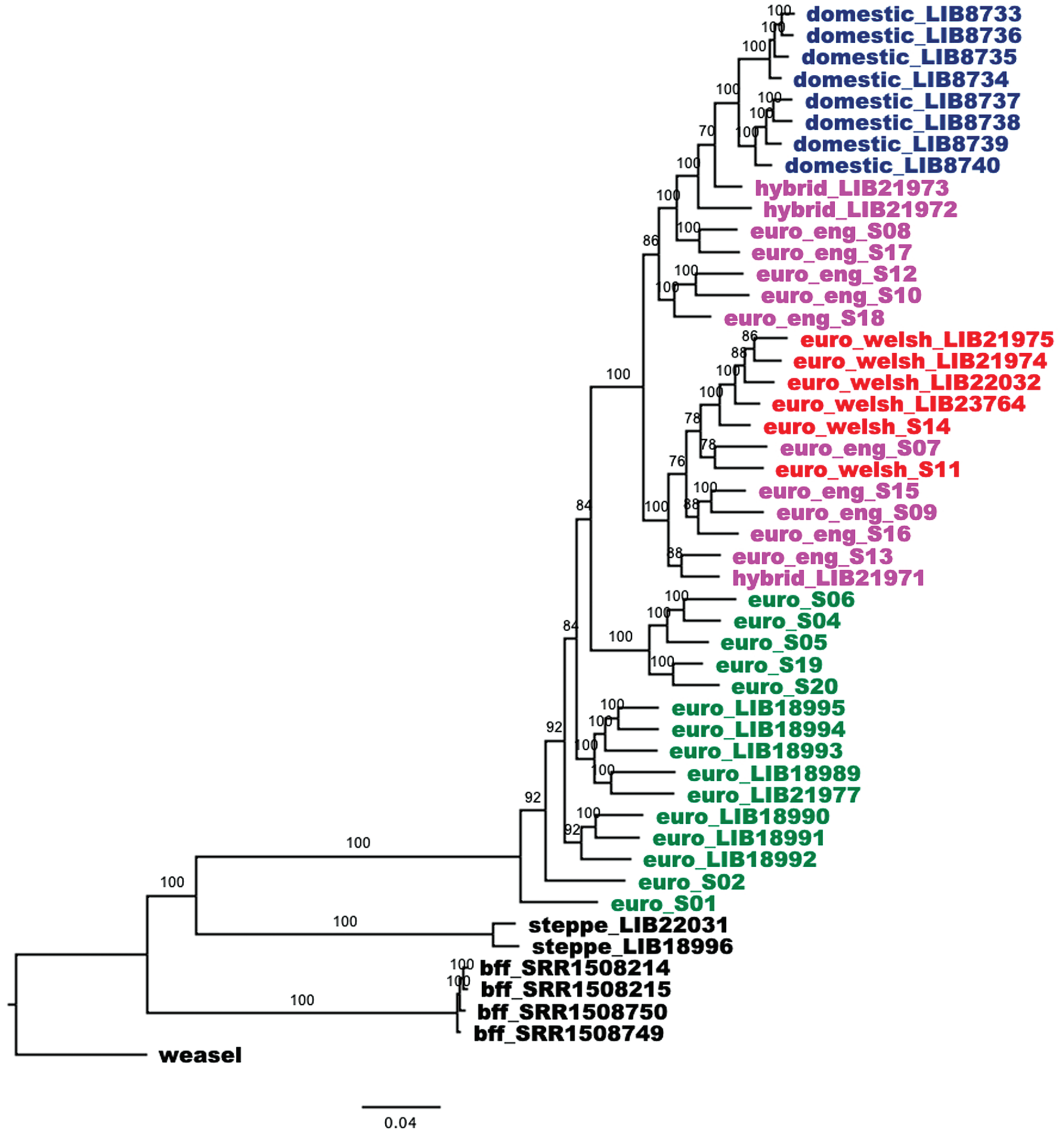
ML phylogeny with 1,000 bootstrap replicates, for genome-wide SNPs of all 49 samples. Numbers at nodes refer to bootstrap support values of 95 and above. Taxa are labeled as in Fig. 2. The tree is rooted with Least Weasel and branch lengths are in expected substitutions per site.

We used RAxML to create ML phylogenies for the whole mitochondrial genomes (Fig. 2) and genome-wide SNPs (Fig. 3) (Stamatakis 2014). In both phylogenies Steppe polecats and Black-footed ferrets form distinct clades, with Steppe polecat forming a sister clade to the European polecats. Also in both phylogenies, European mainland polecats form a paraphyletic group and there is a separate clade for domestic ferrets and British polecats and hybrids, the structure of which varies between the 2 phylogenies.

In the mitochondrial phylogeny (Fig. 2), all but one of the Welsh polecats form a clade with 3 English polecats (all from western England), with the remaining British polecats, domestic ferrets, and phenotypic hybrids forming a separate clade. This suggests 2 distinct mitochondrial haplotypes within Great Britain; one a Welsh polecat haplotype, unique from that found in mainland Europe, the other a domestic ferret haplotype. The multiple sequence alignment showed 78 out of 15,511 sites segregated all samples of the Welsh polecat haplotype from all samples of the domestic ferret haplotype.

In the genome-wide SNP phylogeny (Fig. 3), British polecats are separated into 2 distinct clades—1 containing domestic ferrets, 2 of the 3 hybrids, and 5 English polecats, the other containing all the Welsh polecats, along with 5 English polecats and the remaining hybrid. This clade also separates into 2 subclades, 1 containing all the Welsh polecats (with the addition of 1 English polecat, S07) and the other containing all remaining English polecats and hybrids. The 3 samples of English polecats that cluster with the Welsh polecats in mitochondrial tree (samples S10, S12, and S18) are all found with the domestic ferret branch of the genome-wide SNP tree but form a separate clade at the base.

#### European mainland polecats

Polecats from the European mainland also cluster by geographical region with samples originating from Austria, Germany, Italy, France, and Spain. (Supplementary Table S1).

In the mitochondrial genome tree (Fig. 2), polecats from Austria and Italy cluster into well-defined clades, whilst the remaining samples from Germany, France, and Spain are less well defined. The strongest subpopulation structure is observed in the genome-wide SNP phylogeny (Fig. 3). Samples from Austria and Italy form separate clades, along with a clade with samples from Spain and France, which are then separated into separate subclades of Spanish and French samples. Finally, the samples from Germany form nested distinct lineages at the base of the European polecat samples.

**Fig. 4.**
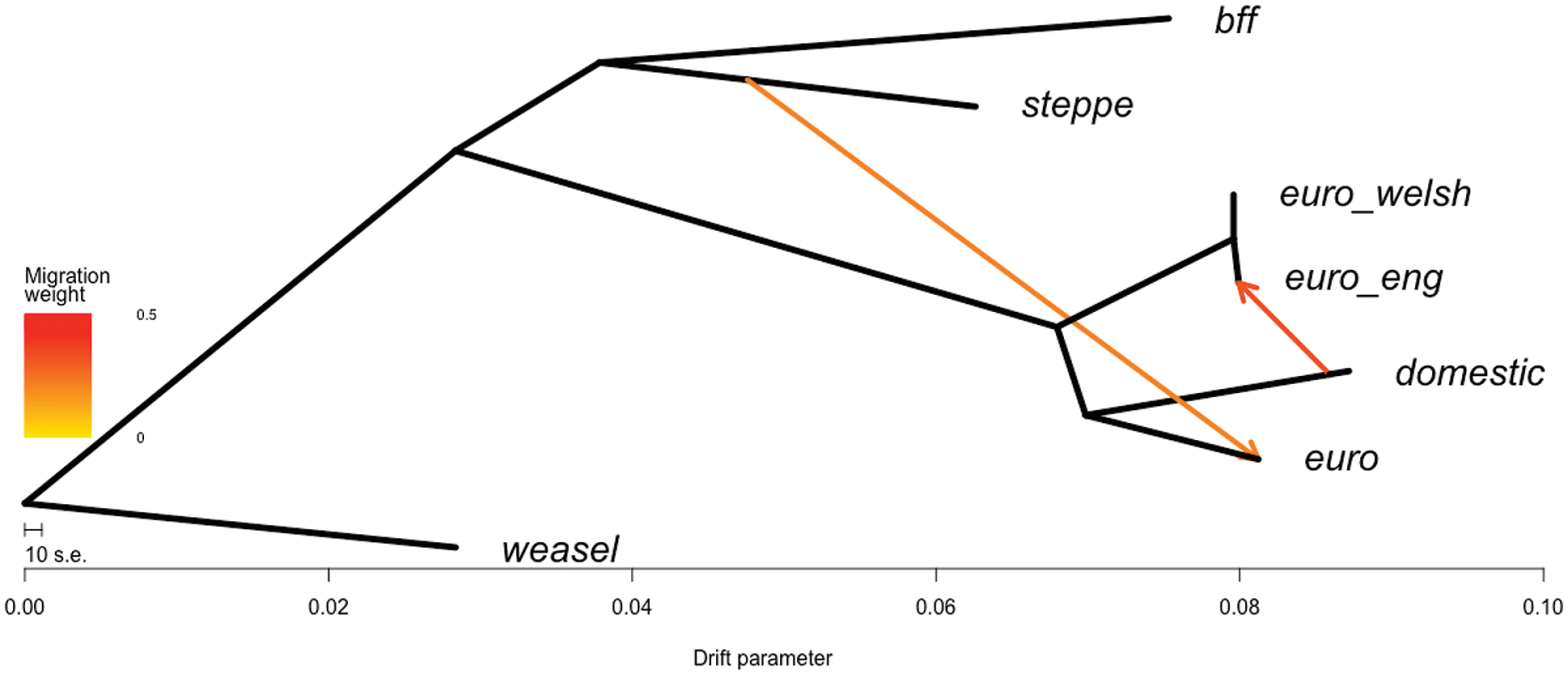
TreeMix phylogeny showing the optimum number of migration edges (2). The tree is rooted by Least Weasel (“weasel”). Populations are labeled as in Fig. 2. Migration arrows are colored according to their weight. Horizontal branch lengths are proportional to the amount of genetic drift that has occurred along the branch.

We used TreeMix to examine population splits and migration events (Pickrell and Pritchard 2012), and calculated that the optimum number of migration edges as 2 (Fig. 4, Supplementary Fig. S1). As is seen in the nuclear ML phylogeny (Fig. 3), Steppe polecat and Black-footed ferret form sister species, with mainland European polecats forming a single clade and British polecats and ferrets forming another clade. There is a strong migration edge from domestic ferret (“domestic”) to English polecat (“euro_eng”), suggesting gene flow between them, along with another migration edge (of a smaller migration weight) from Steppe polecat (“steppe”) to mainland European polecat (“euro”).

### Population structure

**Fig. 5.**
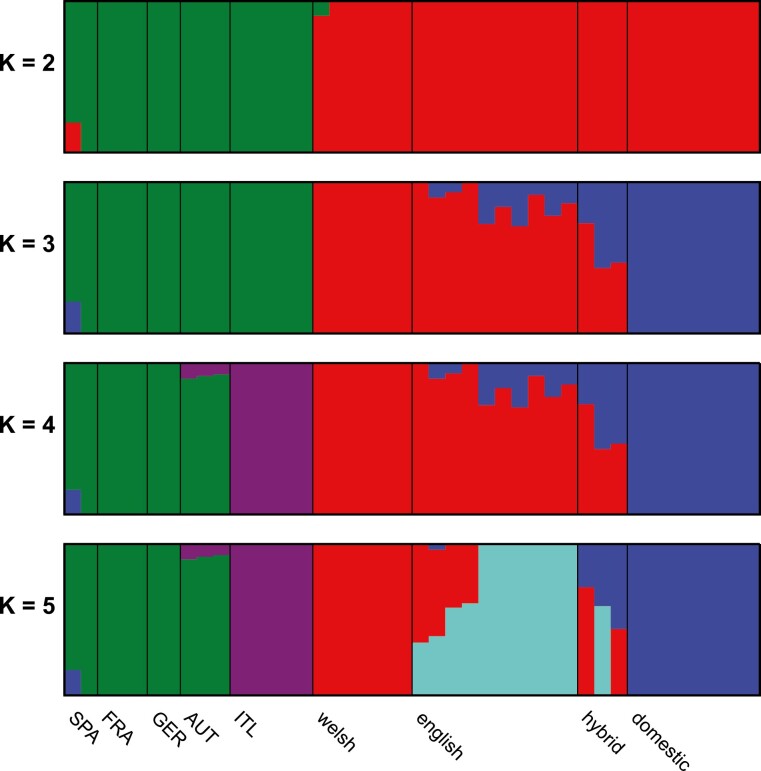
Admixture plots for values of *K* = 2 (top) to *K* = 5 (bottom). Statistical support gives *K* = 3 as the most significant number of populations. Samples of European polecat from the European mainland have been further broken down into their country of origin as follows. SPA = Spain, FRA = France, GER = Germany, AUT = Austria, and ITL = Italy.

We used Admixture to plot population structure across 5 values of *K* (1–5) (Fig. 5) and calculated the CV error for each value of *K*. CV error increased noticeably after *K* = 3. We included phenotypic polecat × ferret hybrids in the plots as a separate group to demonstrate the differences (and similarities) to those individuals that had been phenotyped as “pure” European polecats. At least one of the hybrids has more polecat-like genetic structure (0.729) than some of the phenotypically pure English polecats (0.709 to 0.999), most of which show ferret-like genetic structure to a varying degree (Supplementary Table S3). Additionally, there is evidence of ferret introgression in 1 sample of European mainland polecats from Spain. Also, there is a suggestion of a unique genetic structure in the Italian population of polecats, albeit at *K* values (*K* = 4 and *K* = 5) higher than the most significant (*K* = 3).

#### Nucleotide diversity

We calculated nucleotide diversity (*π*) for each population of polecat, along with 2 outgroups (Steppe polecat and Black-footed ferret) and domestic ferret to compare the genetic diversity of the Welsh polecats with that of other polecat populations and other mustelids. As expected, the highly endangered and inbred Black-footed ferret had the lowest value of *π*, followed by that of domestic ferret (Table 1). Welsh polecats sit within the other populations of European polecats, although do not show as much nucleotide diversity as the highest ranking population of Austrian polecats, which have 68% more nucleotide diversity than Welsh polecats. Interestingly, Welsh polecats sit next to Italian polecats, another population which presumably have some degree of isolation since the previous ice age.

**Table 1.** Nucleotide diversity (*π*) for different populations of polecats, along with 2 outgroups (Steppe polecat and Black-footed ferret) and domestic ferret. Numbers in brackets refer to the sample size for each population.

Population (*n*)	*π*
Black-footed ferret (4)	0.000299
Domestic ferret (8)	0.000417
German polecats (2)	0.000474
Welsh polecats (6)	0.000505
Italian polecats (5)	0.000575
Spanish/French polecats (5)	0.000690
Austrian polecats (3)	0.000847
Steppe polecat (2)	0.000937

#### Introgression analyses

The significant results from Hyde identified 4 trios that showed evidence of hybridization. Three out of the 4 trios identify English polecats as hybrids, with the top hit placing domestic ferrets as P1 and Welsh polecats as P2 (Table 2 and Supplementary Table S4).

**Table 2.** Filtered results from HyDe, ordered by *P* value, then *Z*-score. “Gamma” is an indication as to the proportion of genetic loci contributing from P1 and P2, where a value of 0.5 would indicate that on average the hybrid population is 50:50 admixed and where a value of 0.25 would indicate 25% of admixture originating from P2 (although individual gamma scores may vary around the average).

P1	Hybrid	P2	*Z*-score	*P*	Gamma
Domestic	euro_eng	Welsh	159.040951	0	0.33624814
Euro	euro_eng	Welsh	38.0325902	0	0.05330685
Euro	domestic	Welsh	28.7314146	0	0.08208683
Domestic	euro_eng	Euro	4.96293934	3.48E−07	0.98900496

We then tested for hybridization at the individual level within the populations that have significant levels of hybridization (Supplementary Table S5). Table 3 shows individuals across the hybrid population of the 4 trios listed in Table 2 that have a Gamma value >0.2 and <0.8. The only individuals that met this requirement were the English polecats where P1 and P2 were domestic ferret and Welsh polecats respectively. Additionally, we noted that despite being phenotyped as a hybrid, individual hybrid_LIB21971 shows less evidence of hybridization than 7 English polecats which were all phenotyped as “pure” polecats.

**Table 3.** Statistically significant HyDe tests for hybridization on individuals of hybrid populations flagged as significant in Table 2 and that have a Gamma value >0.2 and <0.8.

P1	Hybrid	P2	*Z*-score	*P*	Gamma
Domestic	hybrid_LIB21973	Welsh	200.10	0	0.533688723
Domestic	hybrid_LIB21972	Welsh	191.14	0	0.569239275
Domestic	eng_euro_S17	Welsh	186.55	0	0.422036059
Domestic	eng_euro_S10	Welsh	149.59	0	0.372189884
Domestic	eng_euro_S18	Welsh	158.44	0	0.350551109
Domestic	eng_euro_S08	Welsh	164.65	0	0.346368603
Domestic	eng_euro_S12	Welsh	129.86	0	0.298052044
Domestic	eng_euro_S07	Welsh	130.62	0	0.275299731
Domestic	eng_euro_S09	Welsh	136.08	0	0.266102112
Domestic	hybrid_LIB21971	Welsh	133.00	0	0.256771089
Domestic	eng_euro_S13	Welsh	123.43	0	0.234519286
Domestic	eng_euro_S16	Welsh	116.13	0	0.231423632
Domestic	eng_euro_S15	Welsh	104.01	0	0.208069401

To examine introgression in the context of gene flow between populations, we used Dsuite to calculate the *D*-statistic for the same combination of trios used in the HyDe analyses above (Malinsky *et al.* 2020). The output from *Dsuite Dtrios* echoed that of the filtered HyDe results in that the domestic ferret and English polecats showed the highest amount of gene flow (Table 4). The 2 highest ranking trios involved gene flow between English polecats and domestic ferrets, with European mainland polecats or Welsh polecats as P1.

**Table 4.** Output of Dsuite Dtrios. The combination of P1, P2, and P3 are ordered so as the *D*-statistic is always positive and all the results, including the *f*4-ratio reflect evidence of excess allele sharing between P3 and P2 for each trio.

P1	P2	P3	*D*-statistic	*Z*-score	*P* value	*f*4-ratio
Euro	English	Domestic	0.322879	27.8886	0	2.01083
Welsh	English	Domestic	0.18104	20.112	0	1.57156
Euro	Welsh	Domestic	0.196827	16.9025	0	1.26856
Welsh	English	Euro	0.0345665	6.92304	2.21E−12	0.12141

**Fig. 6.**
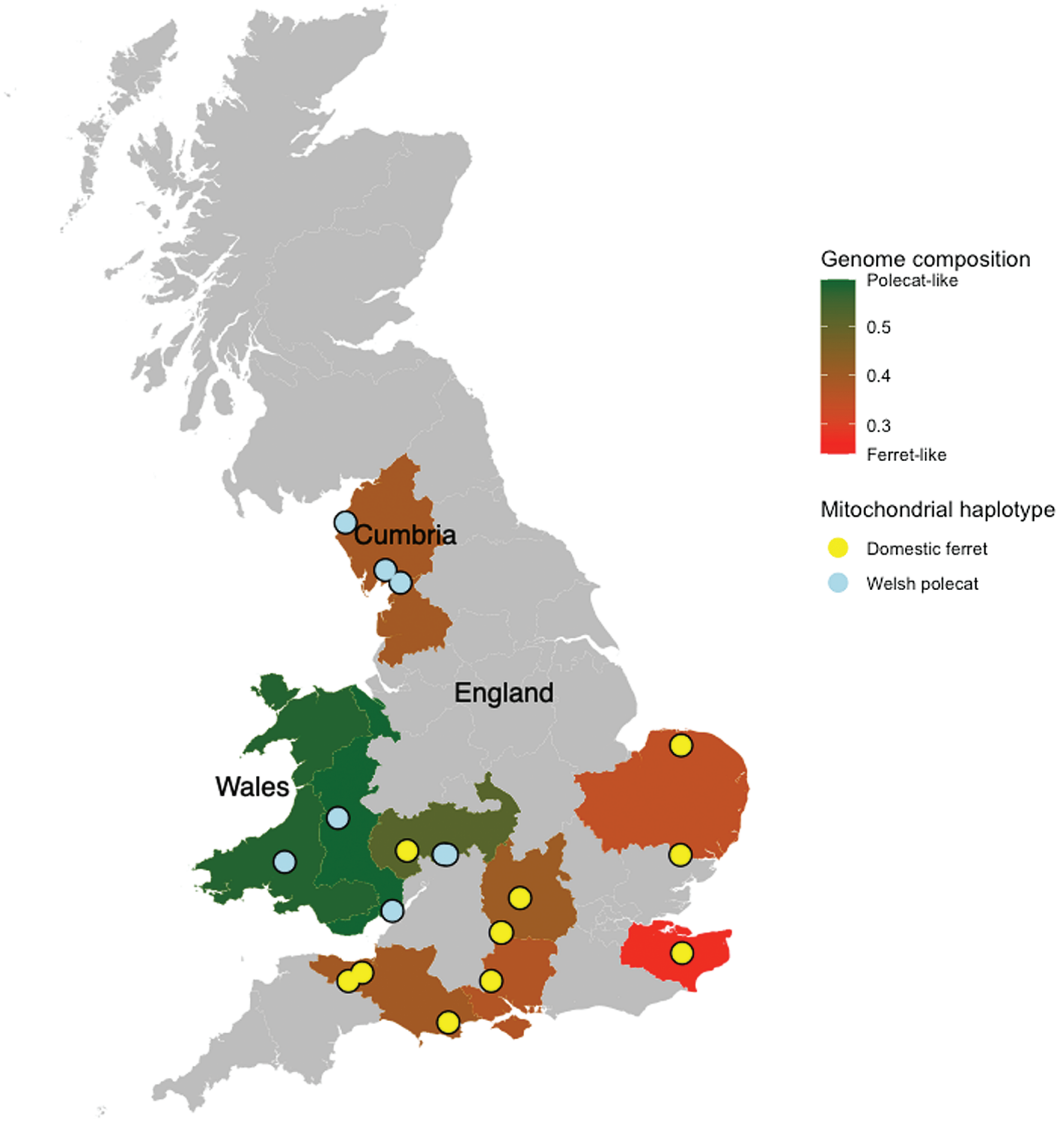
Distribution of polecat- and ferret-specific alleles across samples of European Polecats in Great Britain, along with mitochondrial genome haplotypes assigned in Fig. 2. Locations named on map refer to major areas mentioned in text.

We identified 15,755 sites that were homozygous reference in 8 high-coverage ferret genomes (ferret-specific alleles) and homozygous alt (all to the same alt allele) in the 7 high-coverage European mainland polecats (polecat-specific alleles). We calculated the number of ferret- and polecat-specific alleles in each British polecat sample and mapped the distribution of ferret- and polecat-specific alleles across Great Britain, along with the sample-specific mitochondrial haplotypes, assigned in Fig. 2 (Fig. 6). We also calculated the FDR of this method on a 12 Mb capture array across 76 domestic ferret samples. Across the 76 samples 2,382 genotype were called, 2,362 of which were called as homozygous ref (ferret-specific alleles), giving an FDR of 0.0008, providing high confidence that the number of ferret-specific alleles identified in our 8 whole-genome ferret samples held true across a larger number of samples.

As can be seen in Fig. 6, as the distance increases away from the Welsh refugium, polecats become increasingly ferret-like in their genome composition, with an individual in the south-east of England being the most ferret-like. Regions where polecats persisted during the 1900s bottleneck (Wales and English counties bordering Wales) and regions where Welsh polecats were reintroduced (Cumbria), still show predominantly polecat mitochondrial haplotypes (the exception being sample LIB22032), whilst all other samples have ferret mitochondrial haplotypes producing a clear division in haplotypes. Polecats from mainland Europe show the greatest genetic identity to those in Wales, followed by central western England and Cumbria.

#### Topology weighting analysis

**Fig. 7.**
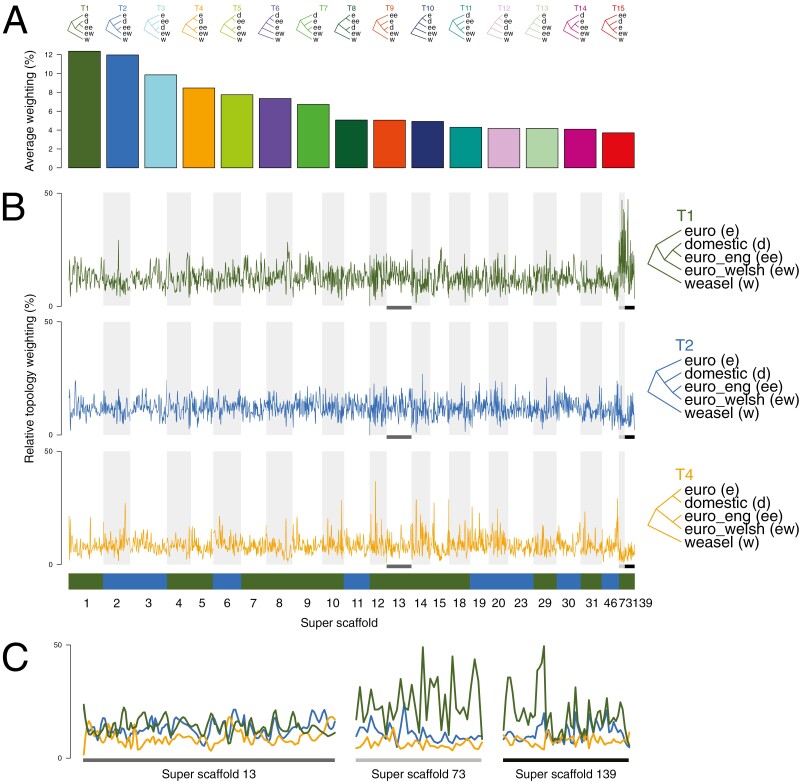
TWISST analyses of domestic ferret and polecat populations along the 23 longest scaffolds, and scaffolds associated with putative adaptive introgression (A) shows the genome-wide average weighting of the 15 possible topologies with the 5 specified population. (B) shows the weighting of the 2 most frequent topologies, as well as the topology indicated by TreeMix analysis, across the scaffolds. The color blocks below the panels show which topology out of the 15 was the most frequent overall across each scaffold, using the colors shown in panel A (all are either T1 or T2). The *y*-axis gives the relative frequency at which a topology is inferred across the linkage groups (*x*-axis). (C) shows the 3 super-scaffolds putatively associated with adaptive introgression in more detail, with all 3 topologies from B plotted. The gray or black × axis bars correspond to the highlighted sections in B. The *y*-axis in C is the same as B. w, weasel; e, mainland European polecat (euro on panel B); d, domestic ferret (domestic on panel B); ee, English polecat (euro_eng on panel B); ew, Welsh polecat (euro_welsh on panel B).

The topology weightings out of the 15 possible topologies of the 5 populations (weasel, Welsh polecats, English polecats, mainland European polecats and domestic ferrets) were compared across the longest 23 assembled scaffolds of the genome assembly (Fig. 7). The most frequent topology shows the domestic ferret as most closely related to the English polecats. The next most frequent shows the English and Welsh polecats as sister to each other, with the domestic ferret most closely related to this group (Fig. 7A). The genome-wide distribution of the latter topology shows a relatively equal range of distribution across the genome (Fig. 7A and B, middle panel in blue), whereas the first topology shows distinct peaks in super-scaffolds 73 and 139 (Fig. 7B, upper panel in green). A topology similar to that inferred by TreeMix (Fig. 4), where the domestic ferret is closest related to the European mainland polecat, is only the fourth most frequent topology (Fig. 7A), and is fairly uniform across the genome, with a few distinct short spikes (e.g. Super-scaffolds 12, 46, and 73).

### Adaptive introgression

From our analyses above, it is apparent that English polecats show introgression from hybridization between domestic ferrets and Welsh or European mainland polecats. Given the geographic limitations of our populations, we used Dsuite Dinvestigate to examine adaptive introgression in English polecats (P2), using Welsh polecats as P1 and domestic ferrets as P3 and selected the top 1-percentile of windows (51 windows) that had positive *f*_dM_ values, suggesting gene flow between English polecats and domestic ferrets. We also identified 148 Fst outliers between domestic ferret and Welsh polecats (identified in the HyDe analyses as the parental populations of the English polecat hybrids), all of which overlapped 7 of the 51 introgressed windows. The 7 windows spanned 3 scaffolds and composed either a single window within a scaffold or multiple overlapping windows forming a single continuous window in that scaffold (i.e. all 3 scaffolds contained only 1 continuous introgressed region) (Table 5 and Fig. 7C). Across the 3 scaffolds, these windows contained 2 protein-coding genes in the domestic ferret genome (ENSMPUG00000001059 and ENSMPUG00000008056), both of which are associated with cognitive function and sight in humans.

**Table 5.** Intersection of the top 1% introgressed loci and the top 1% Fst outliers showing the number of intersections per scaffold, along with the combined span of the windows, the number of Fst outliers found within those windows, and the number of protein-coding genes that intersected both the windows and Fst outliers.

Scaffold name	N. of introgressed windows	Total span of introgressed windows	Fst outliers	N. protein-coding genes
Super-Scaffold_13	1	309 kb	140	1
Super-Scaffold_73	3	4.2 Mb	4	1
Super-Scaffold_139	3	1.6 Mb	4	0

**Fig. 8.**
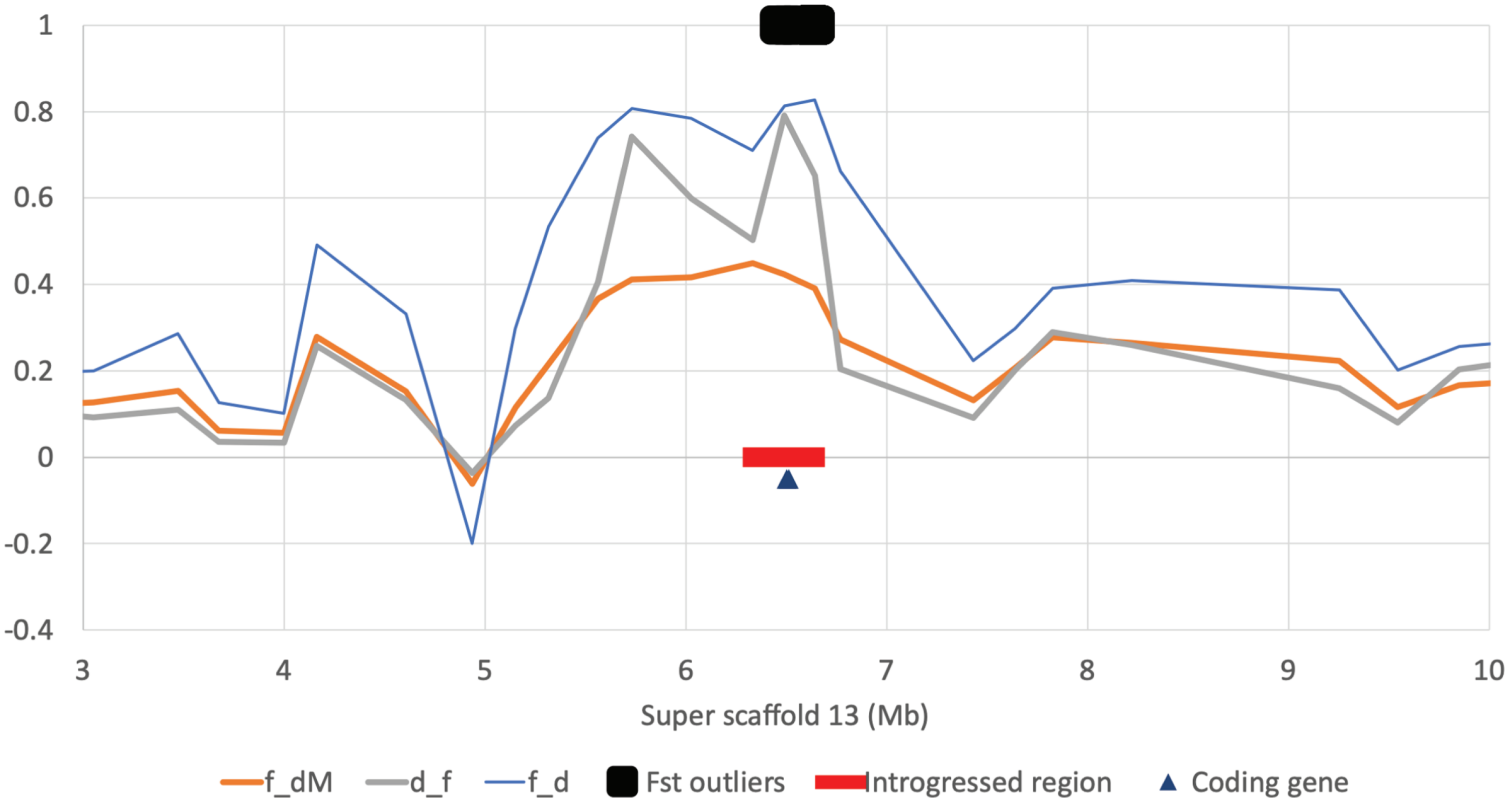
Admixture statistics for *f*_d_, *f*_dM_, and df across a 7 Mb stretch of Super-scaffold_13. The red bar highlights the window of 1,000 SNPs in the top 1-percentile of *f*_dM_ values, the black rectangle represents a cluster of 140 Fst outliers, and the black triangle represent the location of the protein-coding gene ENSMPUG00000008056.

Of the total 148 Fst outliers, 140 of them occur within one 309 kb window on Super-Scaffold 13 (Table 5 and Fig. 8). Within this region there is 1 protein-coding gene, ENSMPUG00000008056. The human ortholog of this gene, C19orf12, encodes for a mitochondrial associate transmembrane glycine zipper that is thought to act as a regulatory protein for magnesium transporters. Mutations in this gene are associated with a range of conditions, such as neurodegeneration with brain iron accumulation (NBIA), progressive movement disorders, spasticity, neuropathy, cognitive dysfunction, and optic nerve atrophy (Hartig *et al.* 2011; Landoure *et al.* 2013; Venco *et al.* 2015; Dusek *et al.* 2020; Espinos *et al.* 2020). It was also recently identified as a top-ranked gene associated with performance in a spatial cognitive task in Mountain Chickadees (Branch *et al.* 2022).

The other coding gene, ENSMPUG00000001059, found on Super-Scaffold_73 (Supplementary Fig. S2), is orthologous to the human CNTNAP5 gene, one of the contactin-associated proteins (Caspr), that participate in nerve excitation and conduction, along with neurotransmitter release in myelinated axons. Mutations in CNTNAP5 are associated with autism and neurodevelopmental disorders (Aleo *et al.* 2020; Ludington *et al.* 2020; Narita *et al.* 2020) as well as glaucomatous neurodegeneration (Chakraborty *et al.* 2021).

Finally, although no protein-coding genes intersect with the Fst outliers on Super-Scaffold_139 it should be noted that ENSMPUG00000010497, orthologous to the Human RP1 axonemal microtubule-associated gene, is located less than 15 kb downstream from the Fst outliers. RP1 is a key gene in the formation of the outer segment of rod and cone photoreceptors in the eye (Goldberg *et al.* 2016). Mutations in RP1 are a common cause of retinitis pigmentosa, which involves a breakdown and loss of cells in the retina (Liu *et al.* 2004).

## Discussion

### British polecat introgression

Hybridization and introgression may increase the adaptive potential of species by the fixation of beneficial alleles and can be used as a tool for species recovery (Chan *et al.* 2019; Quilodran *et al.* 2020). Whole-genome sequencing has allowed quantitative assessment of introgression in British polecats by allowing the use of new tools and methods developed around whole-genome SNP data. Our analyses show that British polecats, away from the previous refugium of central Wales show varying degrees of introgression with domestic ferrets. Our nuclear phylogeny (using genome-wide SNPs) illustrates 2 clades in British polecats, 1 where some English polecats cluster with domestic ferrets, the other where the remaining English polecats cluster with Welsh polecats (Fig. 3). There are 2 well-supported mitochondrial haplotypes in British polecats—a polecat-like haplotype, confined to Cumbria, Wales and English counties bordering Wales, and a domestic ferret-like haplotype, found across the remaining range (Fig. 2). Previous work suggested the presence of 2 mitochondrial haplotypes in British polecats, one found in Cumbrian polecats, the other in Welsh polecats (Costa *et al.* 2013). We found no evidence to support this, with samples from both areas occurring within the same clades and no segregating SNPs between samples from the 2 proposed groups, supporting that found by (Davison *et al.* 1999) (although [Costa *et al.* 2013] had a greater number of samples than in our study [169 samples classified as polecat or hybrid]). The placements of British polecats across the 2 mitochondrial clades are inconsistent with those in the nuclear phylogeny, suggesting a high degree of hybridization and introgression in British polecats outside Wales.

Additional evidence supporting introgression within British polecats can be seen in the TreeMix analysis, which allows both population splits and migration events, and shows a high migration weight between English polecats and domestic ferret (Fig. 4). ADMIXTURE analysis further demonstrates introgression between domestic ferrets and English polecats. Additionally, it shows that many of the English polecats phenotyped as “pure” polecats, show close to, or in some cases, more introgression than those phenotyped as hybrids.

Further analyses using HyDe (Tables 2 and 3) and Dsuite (Table 4) shows that the most likely trios for significant hybridization and introgression are Welsh polecats, domestic ferrets, and English polecats, with English polecats being the introgressed population. Again, in the HyDe analyses an individual phenotyped as a hybrid appears less introgressed than several individuals phenotyped as pure polecats. Finally, 6 samples of English polecats (S07, S09, S13, S15, S16, and hybrid_LIB21971) consistently show lower levels of introgression than the remaining English polecats. They all cluster with the Welsh polecats in the genome-wide SNP phylogeny, have Gamma values of less than 0.3 in the individual Hyde analyses and show the lowest values of ferret genetic structure in the ADMIXTURE analysis.

We have visualized the distribution of polecat mitochondrial haplotypes and nuclear alleles across the British range of the polecat (Fig. 6). Polecat mitochondrial haplotypes are restricted to north and central western Britain. It should be noted that all English polecat samples showed some signs of introgression and no “pure” domestic ferrets were found, suggesting very little ongoing ferret introductions. The further away from the 1900s central Wales refugium polecats occur, the more ferret-like their genomes become, consistent with previous work suggesting that male polecats expand their range away from the refugium and hybridize with female feral domestic ferrets (Davison *et al.* 1999; Lode 2001; Raymond *et al.* 2021). A similar pattern of male dispersal is found in other European carnivores such as the Eurasian lynx (*Lynx lynx*) (Herrero *et al.* 2021), Scandinavian brown bear (*Ursus arctos*) (Waits *et al.* 2000), and Red fox (*Vulpes vulpes*) (Gachot-Neveu *et al.* 2009).

Looking at the genome in more detail, the TWISST analyses show that considering English polecats as a separate population, most regions of their genomes show signs of introgression with domestic ferret, although small windows of genome with no apparent introgression occur (i.e. where T4 in Fig. 7B is the dominant topology, e.g. the small spike in Super-scaffold 12). The introgressed regions of English polecat’s genomes contain 2 (and possibly 3) genes that are associated with cognitive function and sight in humans, although the function of these genes in mustelids cannot be automatically inferred and as with any analyses of this type should be viewed with caution.

In Eurasia, domesticated mammals hybridizing with their wild ancestors has been noted in several species. For example, hybridization has been found between domestic pig and wild boar (Giuffra *et al.* 2000; Goedbloed *et al.* 2013), modern domestic sheep and wild-living Soay sheep (Feulner *et al.* 2013), domestic goat and Alpine ibex (*Capra ibex*) (Grossen *et al.* 2014), and the Tibetan Mastiff and Gray Wolf (*Canis lupus*) (Miao and Wang 2017), with many of these introgression events being related to adaptation (Fontsere *et al.* 2019).

### Welsh polecats

British polecats underwent a significant population decline and range contraction to the point that they were restricted to unmanaged forests in central Wales. This population decline does not appear to have restricted the amount of genetic diversity to the point that it has less diversity than other polecats from the European mainland (Table 1). We find evidence of genetic divergence between polecats in Wales to those found on the European mainland. In the mitochondrial genome and whole-genome phylogenies, Welsh polecats never cluster with European mainland polecats (Figs. 2 and 3), they form a distinct cluster in the Admixture analysis and when *K* is lowered to 2 clusters, Welsh polecats cluster with the same group that includes domestic ferret, rather than mainland European polecats (Fig. 5). Additionally, when we examine the distribution of polecat-specific alleles across Great Britain (Fig. 6), although Welsh polecats show the greatest number of polecat-specific alleles, this proportion does not reach 1.0, but reaches only ~0.6, although it should be noted that this method does not take into consideration alleles unique to only Welsh polecats. Several factors may be involved here. Historical geographical isolation may have led to it becoming genetically distinct, to the point where it represents a distinct species. Additionally, due to its closer placement to domestic ferret it could be argued that the Welsh polecat is the ancestral population of the domestic ferret, although the (somewhat vague) literature suggests that this occurred elsewhere. Finally, it could be possible that Welsh polecats have low levels of ferret introgression brought about by recent hybridization. These factors pose important conservation challenges. If introgression with ferret has occurred, a number of questions could be asked. Is this introgression ongoing and how is it driven? How recent was the introgression (i.e. before or after the recent bottleneck)? Are there any “pure” polecats remaining in Great Britain and how do we genetically identify them? Whichever elements are involved, more work needs to be carried out on the taxonomic status and demographic history of this population. “Unofficial” releases of polecats have been made into areas such as Cumbria as well as Argyll and Perthshire in Scotland. These releases are thought to be of captive stock of unknown genetic purity (Birks and Kitchener 1999; Birks 2015). The release of polecats of unknown origin exacerbates the picture of polecat diversity, so the ability to identify pure polecats is essential to conservation efforts that involve translocation and release schemes.

### European mainland polecats

Although not the main focus of this work, the phylogenetic analyses provide clear evidence that the ancestral species of the domestic ferret is the European polecat and not Steppe polecat. In all phylogenetic work (Figs. 2–4), the European polecat is always the closest basal species to the domestic ferret, suggesting that domestic ferrets originated from European polecats. We also see evidence of introgression between European polecat and Steppe polecat (Fig. 4), supporting further evidence of hybridization between the 2 species, but it should be noted that the origin of Steppe polecats used in this work (Inner Mongolia) is well beyond the sympatric range overlap with European polecats that earlier work has studied (Cserkész *et al.* 2021; Szatmari *et al.* 2021), suggesting the possibility of widespread introgression.

The phylogenetic and Admixture analyses suggest genetic structure across the European polecats. Polecats from Austria and Italy cluster into well-defined clades in both the mitochondrial and genome-wide SNP phylogenies, and the Italian polecats are further allocated a separate genetic group in the Admixture analyses when *K* is increased to 4 (although *K* = 3 is the most significant value). These results suggest a well-defined genetic structure and that European polecats in Italy may form a separate phylogenetic unit from those on the rest of mainland Europe, driven by historical geographical separation between the 2 populations during the previous ice age (Hewitt 1996). Historical glaciation has been the driving force for the divergence of a number of species in Italy, including the Italian Hare (*Lepus corsicanus*) (Alves *et al.* 2008), Calabria pine vole (*Microtus brachycercus*) (Galleni *et al.* 1994), Calabrian black squirrel (*Sciurus meridionalis*) (Wauters *et al.* 2017), Calabrian forest dormouse (*Dryomys aspromontis*) (Bisconti *et al.* 2018), and Apennine shrew (*Sorex samniticus*) (Yannic *et al.* 2008).

#### Summary

For the first time, we have carried out population-level whole-genome sequencing on European polecats. Our analyses show that English polecats are highly introgressed with domestic ferrets, showing geographically separated mitochondrial haplotypes, with polecats becoming increasingly introgressed with domestic ferrets as they move further away from the 1900s polecat refugium. Phenotyping polecats as “pure” or “hybrid” is not accurate, with all samples of English polecat, regardless of phenotype showing some degree of introgression, and with those assigned as “hybrid” sometimes being less introgressed than those assigned as “pure.” Introgression is distributed widely across the genome and introgressed regions with Fst outliers contain genes associated with cognitive function and sight. Polecats found in Wales appear to be a separate genetic lineage from those found on the European mainland, but more work is needed to assess the demographic history of Welsh polecats.

## Supplementary Material

esac038_suppl_Supplementary_Material

esac038_suppl_Supplementary_Table_S1

esac038_suppl_Supplementary_Table_S2

esac038_suppl_Supplementary_Table_S3

esac038_suppl_Supplementary_Table_S4

esac038_suppl_Supplementary_Table_S5

esac038_suppl_Supplementary_Table_S6

## Data Availability

The domestic ferret reference genome used in this study can be found under ENA accession number GCA_920103865. Read data for the 4 Black-footed ferret samples can be found at NCBI under BioProject PRJNA254451. Read data for the remaining samples can be found under ENA Study accession PRJEB48359 and described in detail in Supplementary Table S6.
