# Supplementary material for: Extensive genome introgression between domestic ferret and European polecat during population recovery in Great Britain
Source: J Hered. 2022 Aug 6;113(5):500–15. doi: 10.1093/jhered/esac038 (PMC9584812; doi:10.1093/jhered/esac038)
Supplement: esac038_suppl_Supplementary_Material [file esac038_suppl_supplementary_material.pdf]

Etherington_SupMat_Figures

Polecat introgression. JHered

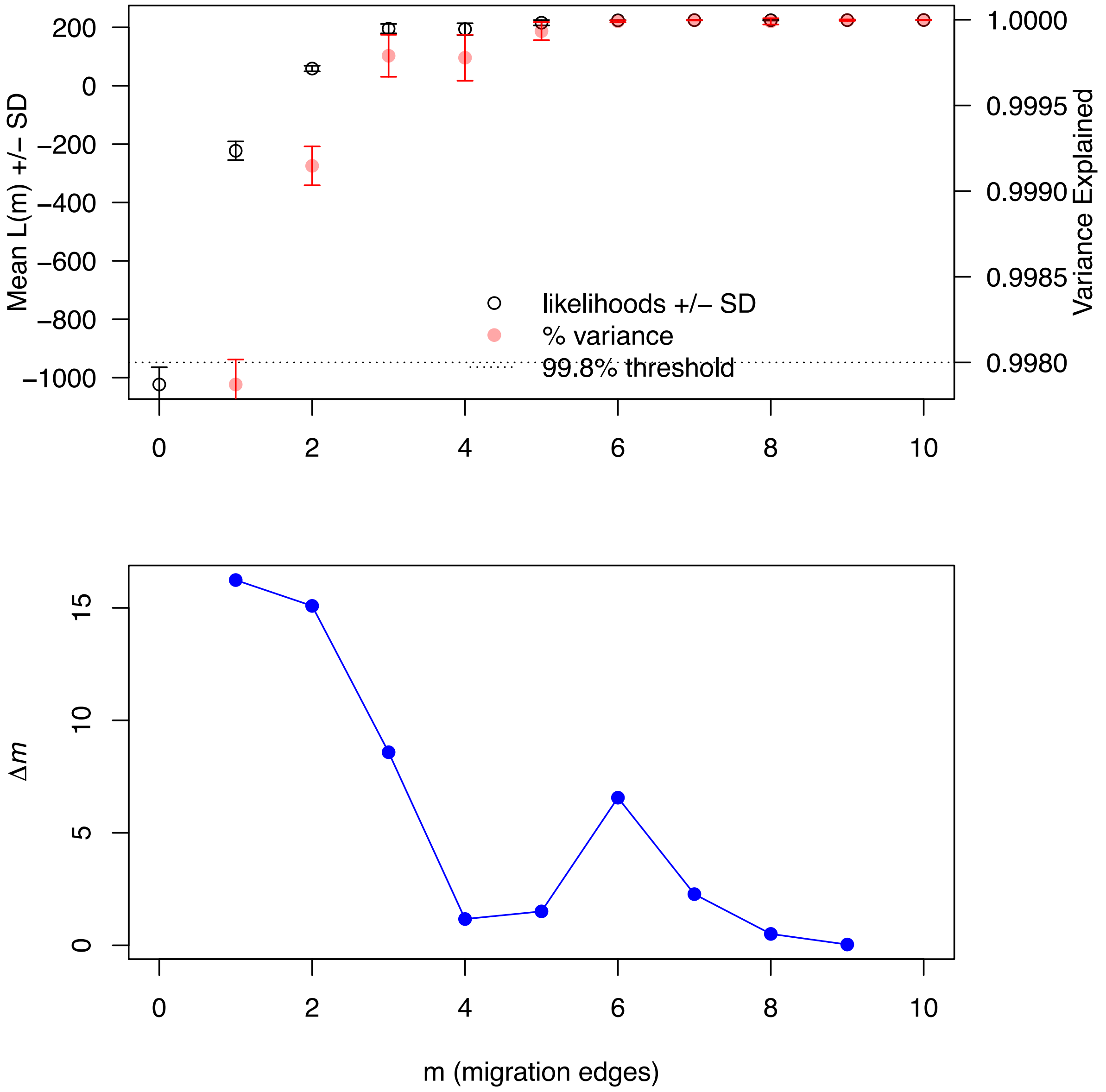

Supplementary Figure S1. Estimation of the optimal number of migration edges in TreeMix using the R package OptM with the default Evanno method. The number of migration edges

(2) was chosen based on a plateau of log likelihoods and when greater than 99.8% of the variance was explained.

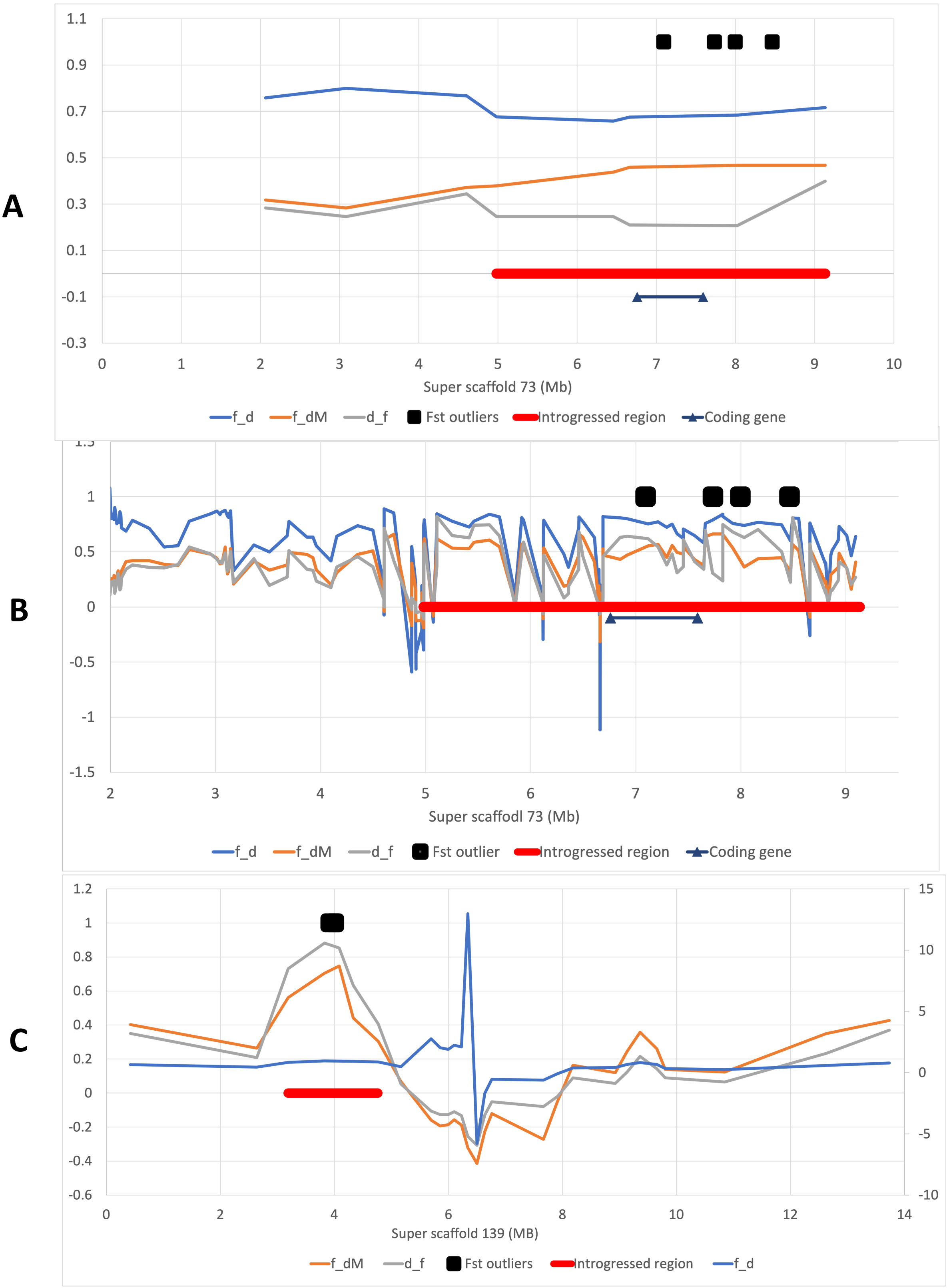

Supplementary Figure S2. Admixture statistics for f_d, f_{dM}, and df across Super-scaffold_73 over A) Windows of 1000 SNPs, and B) windows of 50 SNPs (to view more local variation

across the region), and C) Super-Scaffold_139. The red bar highlights the overlapping windows of 1000 SNPs in the top 1-percentile of f_{dM} values, the black squares represent Fst outliers, and the area between the black triangles (if present) represent the location of the protein-coding genes.

Etherington_SupMat_Tables

Polecat introgression. JHered

Supplementary Table S1. Details of per-sample information. 'Sample Accession ID' refers the ENA sample accession ID, and 'Analyses population' refers to the population (as described in the manuscript) that the sample was allocated.

Supplementary Table S2. Sequencing information for all samples. Bases per sample and read coverage (based on the 2.42Gb domestic ferret genome) refers to post-QC data. Sequenced read-length refers the read-length pre-QC. Samples can be matched to those in Supplementary Table S1 by their Library ID.

Supplementary Table S3. Raw output of the Admixture program, providing genetic partitions for k=3.

Supplementary Table S4. Filtered output of 'run_hyde.ph'. Columns containing only zeros have been removed.

Supplementary Table S5. Output of the 'individual_hyde.ph'. Columns containing only zeros have been removed and an extra column 'Gamma diff' as been inserted to calculate the distance of Gamma (an indication as to the proportion of genetic loci contributing from P1

and P2, where a value of 0.5 would indicate a 50:50 hybrid), from 0.5. The table is sorted by p-value and then Z-score.

Supplementary Table S6. Full details of all ENA accession IDs for samples, experiments, and runs. Samples can be matched to those in Supplementary Tables S1 and S2 by their Library ID.

tree.nwk. Phylogenetic tree in Newick format, specify the relationships between populations in the Dsuite analyses.
